# In Vitro and in Vivo Activity of mTOR Kinase and PI3K Inhibitors Against *Leishmania donovani* and *Trypanosoma brucei*

**DOI:** 10.3390/molecules25081980

**Published:** 2020-04-23

**Authors:** Trong-Nhat Phan, Kyung-Hwa Baek, Nakyung Lee, Soo Young Byun, David Shum, Joo Hwan No

**Affiliations:** 1 Leishmania Research Laboratory, Institut Pasteur Korea, 696 Sampyeong-dong, Bundang-gu, Seongnam-si, Gyeonggi-do 463–400, Korea; trongnhat.phan@ip-korea.org (T.-N.P.); kyunghwa.baek@ip-korea.org (K.-H.B.); 2 Screening Development Platform, Institut Pasteur Korea, 696 Sampyeong-dong, Bundang-gu, Seongnam-si, Gyeonggi-do 463–400, Korea; nakyung.lee@ip-korea.org (N.L.); sooyoung.byun@ip-korea.org (S.Y.B.); david.shum@ip-korea.org (D.S.)

**Keywords:** *Leishmania*, *Trypanosoma*, mammalian target of rapamycin, phosphoinositide 3-kinase, inhibitors

## Abstract

Kinetoplastid parasites, including *Leishmania* and *Trypanosoma* spp., are life threatening pathogens with a worldwide distribution. Next-generation therapeutics for treatment are needed as current treatments have limitations, such as toxicity and drug resistance. In this study, we examined the activities of established mammalian target of rapamycin (mTOR)/phosphoinositide 3-kinase (PI3K) inhibitors against these tropical diseases. High-throughput screening of a library of 1742 bioactive compounds against intracellular *L. donovani* was performed, and seven mTOR/PI3K inhibitors were identified. Dose-dilution assays revealed that these inhibitors had half maximal effective concentration (EC_50)_ values ranging from 0.14 to 13.44 μM for *L. donovani* amastigotes and from 0.00005 to 8.16 μM for *T. brucei*. The results of a visceral leishmaniasis mouse model indicated that treatment with Torin2, dactolisib, or NVP-BGT226 resulted in reductions of 35%, 53%, and 54%, respectively, in the numbers of liver parasites. In an acute *T. brucei* mouse model using NVP-BGT226 parasite numbers were reduced to under the limits of detection by five consecutive days of treatment. Multiple sequence and structural alignment results indicated high similarities between mTOR and kinetoplastid TORs; the inhibitors are predicted to bind in a similar manner. Taken together, these results indicated that the TOR pathways of parasites have potential for the discovery of novel targets and new potent inhibitors.

## 1. Introduction

Neglected tropical diseases including leishmaniasis, human African trypanosomiasis (HAT, or sleeping sickness), and Chagas disease are caused by the pathogenic protozoans *Leishmania* spp., *Trypanosoma brucei* subspecies, and *Trypanosoma cruzi*, respectively. Globally, there are nearly one hundred thousand deaths and over 22 million reported cases associated with these diseases annually [1,2]. There are three types of leishmaniasis: cutaneous leishmaniasis (CL), mucocutaneous leishmaniasis, and visceral leishmaniasis (VL) [3,4]. The disease is distributed throughout 97 countries, mainly in Africa, Asia, and Latin America, but the fatal form of VL mostly occurs in Ethiopia, South Sudan, India, Bangladesh, and Brazil [5,6]. Current treatment options include sodium stibogluconate, which is only available for administration via injection; amphotericin B, which is repurposed from antifungal treatment; and miltefosine, being the only orally administered drug [7]. HAT is caused by protozoa of the subspecies of *T. brucei*, including *T. brucei gambiense* and *T. brucei rhodesiense* in Africa. The disease is usually transmitted by the bite of an infected tsetse fly. It most commonly occurs in rural areas and can be fatal if not treated. The treatment used depends on the type and the stage of infection. Pentamidine or suramin are widely used for the treatment of acute-phase infections. Melarsoprol or eflornithine are usually used for treatment of the second, or neurological, phase of disease [8,9]. In 2009, Drugs for Neglected Diseases initiative developed the oral nifurtimox/intravenous eflornithine combination therapy (NECT) for the second stage HAT, and more recently, in 2019, an effective oral monotherapy of fexinidazole was launch for both disease stages [10,11]. Chagas disease (American trypanosomiasis) is caused by *T. cruzi* and is spread by members of the Triatominae subfamily (kissing bugs); the disease distribution includes Paraguay, Colombia, Venezuela, Argentina, Bolivia, Chile, and Brazil. Symptoms can be developed 10–30 years after infection, and nifurtimox and benznidazole are used to cure infected patients [12,13]. The drugs used to treat kinetoplastid diseases are often highly toxic, expensive, and limited by administration route. These characteristics are significant barriers to their use for treatment of patients in developing countries [14,15,16,17]. Despite an urgent need, the development of next-generation therapeutics has been halted by no or low financial profits, as these diseases occur mostly in the poorest parts of the world [18,19,20]. To reduce the costs and time-consuming safety and efficacy trials, repurposing established drugs used for other diseases is an attractive path to facilitate the development of anti-kinetoplastid therapeutics [21,22,23,24]. 

Target of rapamycin (TOR) is a serine/threonine protein kinase that is a key master regulator of multiple signaling pathways in eukaryotes. The mammalian TOR (mTOR) pathway is involved in various biological functions, such as cellular growth and proliferation, metabolism, survival, motility, and autophagy [25,26,27]. The mTOR protein has a central role in the pathway. It belongs to the family of phosphoinositide 3-kinase (PI3K)-related kinases, which is a protein family with roles in cellular responses to various types of stresses [28,29,30]. Extensive investigations have led to the identification and functional characterization of PI3Kα, β, γ, and δ isoforms; these isoforms are currently considered as attractive targets for new anti-cancer therapies [31,32,33,34]. There are three types of PI3K inhibitors, including dual mTOR/PI3K, pan-PI3K, and isoform-specific inhibitors [35]. Based on the target, more than 35 compounds are being tested in clinical trials for treatment of different types of cancers. Many of these compounds have not advanced to late-phase trials due to toxicity and limitations in activity. However, everolimus, temsirolimus, idelalisib, and copanlisib have been approved by the U.S. Food and Drug Administration for clinical use as cancer treatments [36]. Moreover, ridaforolimus for metastatic soft-tissue sarcoma (clinicaltrials.gov identifier: NCT00538239) and buparlisib for advanced-stage breast cancer (NCT01610284) completed phase III clinical trials. Furthermore, taselisib for breast cancer (NCT02340221), duvelisib for chronic lymphocytic leukemia (NCT02004522), and ipatasertib for advanced-stage breast cancer (NCT03800836) are undergoing phase III clinical trials advocating the potential use of mTOR and PI3K inhibitor for therapeutic interventions [37,38,39].

In *T. brucei,* two orthologues of mTOR, *Tb*TOR1 and *Tb*TOR2, were identified. *Tb*TOR1 is involved in cell cycle regulation and protein synthesis, whereas *Tb*TOR2 coordinates cell polarization and cytokinesis [40,41]. More recently, an unusual TOR exclusively present in kinetoplastids, *Tb*TOR-like 1, was discovered and found to have a role in the control of polyphosphate levels and acidocalcisome maintenance [42]. With the presence of TOR, rapamycin exhibits potent activity against parasite growth via preventing formation of *Tb*TOR complex 2; this mechanism is contrary to the mechanisms discovered in other eukaryotes [40]. Three TORs have been identified for *Leishmania*, and mutant studies revealed that TOR1 and TOR2 are essential for the survival of *Leishmania major* promastigotes, and similar to *T. brucei*, TOR3 is required for acidocalcisome biogenesis [43].

Based on the essentiality of TORs in kinetoplastid parasites, several groups have evaluated the use of mTOR kinase and PI3K inhibitors against the parasites [44,45]. NVP-BEZ235, an anti-cancer agent at the clinical trial stage, is a potent inhibitor of *Leishmania* spp. and *Trypanosoma* spp. in vitro and showed efficacy against *T. brucei* in an animal model [37]. In a CL animal model, it was found that treatment with rapamycin and GSK-2126458 resulted in significant decreases in footpad swelling and parasitemia in the draining lymph nodes in infected animals [38]. These compounds are shown to kill parasites directly. On the other hand, Khadem et al. have demonstrated that selective pharmacological inhibition of the host PI3Kδ with CAL-101 results in significant reductions of parasite burdens in VL and CL animal models [40]. These findings suggest that mTOR/PI3K inhibitors have potential applications for treatment of kinetoplastid diseases. 

In this study, we report the results of an in vitro screening of bioactive compounds against intracellular *L. donovani*. We identified new mTOR/PI3K inhibitors for further assessment for use on different forms of kinetoplastid parasites. Potent compounds in vitro were subjected to in vivo models of VL and HAT. Structural modeling of identified inhibitors binding to the TORs of kinetoplastid parasites were predicted to provide insights on the potential mode of inhibition at molecular level. 

## 2. Results and Discussion

### 2.1. Intracellular Leishmania Pilot Screening

**Figure 1:**
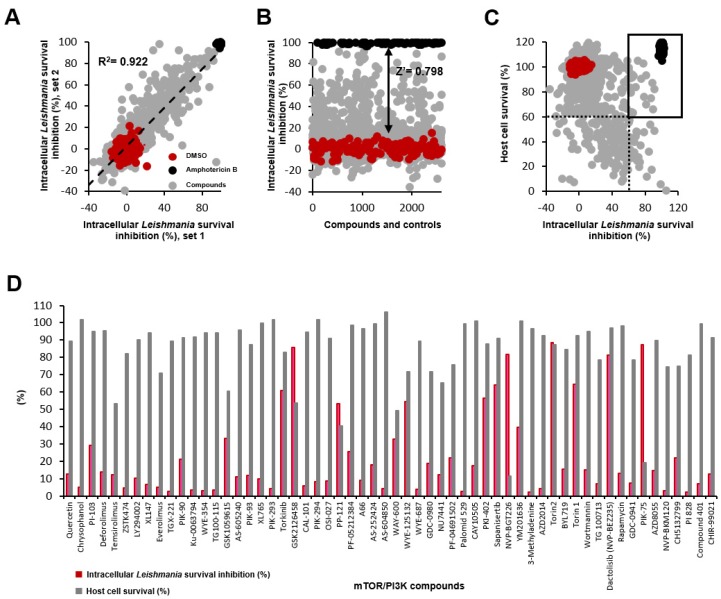
Pilot screening results for intracellular *L. donovani* infection in THP-1 cell line. Red, negative control (DMSO); black, positive control (4 μM amphotericin B); gray, compound. A total of 1742 compounds were screened, with R^2^ = 0.922 (**A**)**,** Z′ = 0.798 (**B**), and host cell survival and intracellular parasite survival inhibition > 60%, black squares (**C**), and cell ratio and intracellular parasite survival inhibition of 58 mammalian target of rapamycin (mTOR)/phosphoinositide 3-kinase (PI3K) compounds (**D**).

We used a high-content screening system to assess a library of 1742 compounds with potential bioactivity against intracellular *Leishmania*. Human acute monocytic leukemia (THP-1) cells infected with *L. donovani* were treated with compounds; images were analyzed to quantify the numbers of parasites and host cells and infection ratios. The reproducibility and robustness of the assay was evaluated by duplicate runs. The correlation between two independent screenings indicated a high degree of linear relationship, with an R^2^ value of 0.922. There were 74 compounds that had activity in set 1 or set 2 (threshold > 60%), and 32 with activity in both sets (Figure 1A). To evaluate assay quality, the Z′ factor was calculated using the average values from duplicate testing. The Z′ factor was given by Z′ = 1 − 3(σ_c_^+^ + σ_c_^−^)/(μ_c_^+^ − μ_c_^−^), where σ_c_^+^/σ_c_^−^ were the standard deviation values of the positive/negative controls and μ_c_^+^/μ_c_^−^ were the corresponding mean values. The Z′ value of the screening using the infection ratio was 0.798; this result indicated excellent assay quality (Figure 1B). 

The screening results indicated that 50 compounds were active, based on a >60% threshold of the inhibition of intracellular parasite survival (Figure 1B). Since this number included the compounds showing activity due to killing of the host cells (low selectivity), we plotted the parasite survival inhibition versus the host cell viability to further filter parasite-selective compounds. With >60% parasite survival inhibition and >60% host cell viability, a final number of 20 compounds was selected out of the 1742 with the overall hit rate of 1.15% (Figure 1C).

### 2.2. Hit Characteristics and Selection of mTOR/PI3K Inhibitor

Among the selected 20 hits, five compounds were in the category of mTOR/PI3K inhibitors. In the screening library, 58 mTOR/PI3K inhibitors were present, and by proportion, 8.62% (= 5/58) was found active within this category of inhibitor. This rate is relatively higher compared to the overall hit rate of 1.15% (Figure 1D). The mTOR complex inhibitors, such as rapamycin and its analogs deforolimus, temsirolimus, and everolimus, were included in the library, but did not show potent inhibition at the screening concentration (10 µM). Previously reported antileishmanial compounds, such as dactolisib (NVP-BEZ235) [37] and GSK2126458 [39], were found active, but the latter one showed some toxicity against the host cell. The compounds known to exhibit activity in vivo via modulation of the host immune response (i.e., CAL-101) had no direct effects on the parasite [38].

**Figure 2:**
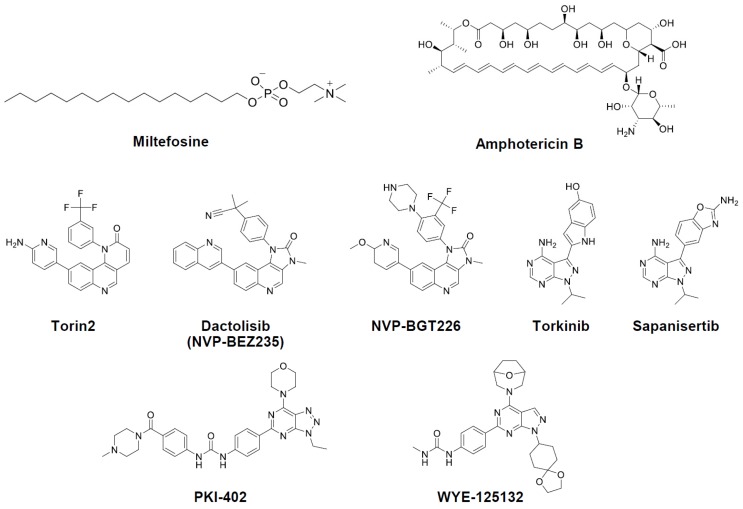
Structures of compounds investigated in this work.

With two additional mTOR/PI3K inhibitors at the border of threshold, a total of 22 compounds were subjected to dose-dilution assays for activity confirmation. In the result, compounds such as dopamine uptake inhibitors (GBR12909 and 3-CPMT), protein kinase A inhibitor (H89), VEGFR inhibitor (cediranib and ENMD-2076), and EGFR inhibitor (afatinib) had either low activity (half maximal effective concentration, EC_50_) or high toxicity (CC_50_). Other compounds, including Y29794, metaphit, Ro 106–9920, SKF 96365, and NNC05–2090 were confirmed to be active, but did not group together into a category of inhibitor. In the case of the mTOR/PI3K inhibitors, all the compounds were well-confirmed with potent activities and sufficient selectivity index (SI) values (Table 1). Based on these results, we sought to focus the subsequent investigations on this class of inhibitors (Figure 2).

### 2.3. In Vitro Activity Against Kinetoplastid Parasite

**Figure 3:**
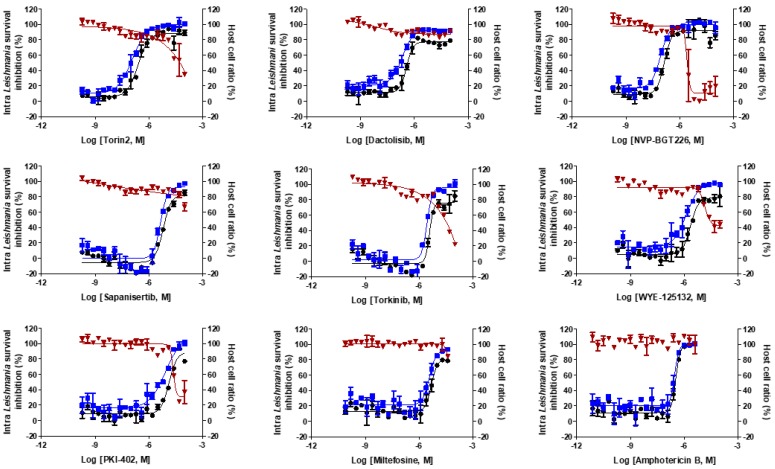
Dose-response curves of miltefosine, amphotericin B, and mTOR/PI3K compounds against Table 1 cells infected with intracellular *L. donovani*. Inhibition infection ratio (%) (● black-filled circles); inhibition parasite number (%) (■ blue-filled squares); and host cell ratio (%) (▼ red-filled triangles). The results are expressed as mean ± standard deviation values for duplicate experiments.

The confirmatory intracellular *Leishmania* assays found that the tested mTOR/PI3K inhibitors had EC_50_ values, ranging from 0.14 to 13.44 μM (Table 1 and Figure 3). The most potent compound was NVP-BGT226 with EC_50_ value of 0.14 μM, which is approximately 2.5 and 30 times more potent than amphotericin B and miltefosine, respectively. The structurally similar compounds, Torin2 (EC_50_ = 0.25 μM) and dactolisib (EC_50_ = 0.36 μM), exhibited activities in the submicromolar range. Torkinib and sapanisertib share the same scaffold, and PKI-402 and WYE-125132 are structurally similar. These inhibitors were moderately potent with EC_50_ values similar to that of miltefosine (Table 1). We then sought to check any relationship between the intracellular *Leishmania* survival inhibition values to mTOR/PI3K inhibition values from published data (Table S2) [46,47,48,49,50]. Interestingly, the inhibitors that are active against mammalian PI3K at low nanomolar range, such as Torin2, dactolisib, and NVP-BGT226, were mostly found active against the parasite except PKI-402.

**Figure 4:**
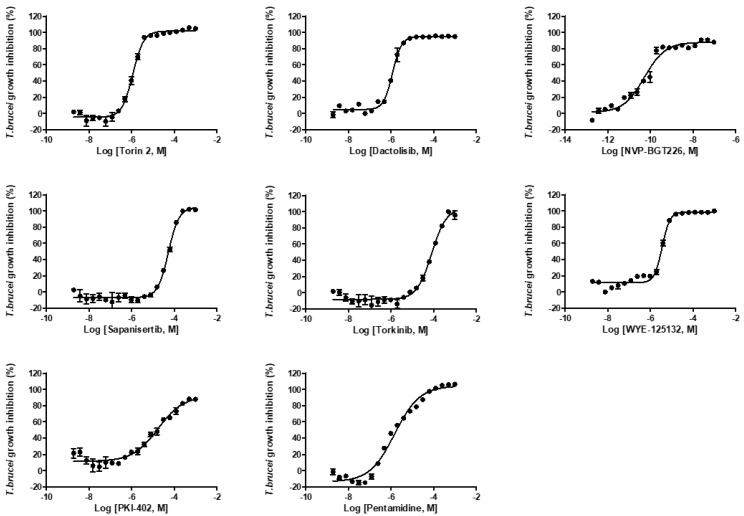
Dose-response curves of pentamidine and mTOR/PI3K compounds against *T. brucei* bloodstream form growth. The results are expressed as mean ± standard deviation values for duplicate experiments.

**Figure 5:**
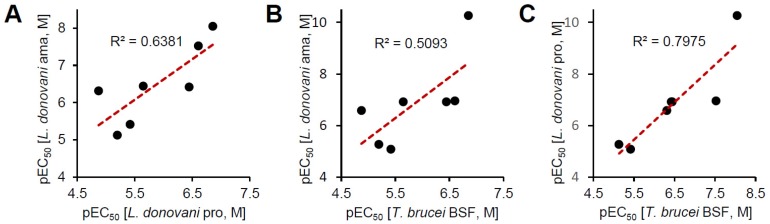
Correlation of pEC_50_ values for seven mTOR/PI3K inhibitors against *L. donovani* and *T. brucei.* (**A**) Correlation of pEC_50_ values against intracellular *L. donovani* and promastigotes. (**B**) Correlation of pEC_50_ values against intracellular *L. donovani* and *T. brucei* BSF. (**C**) Correlation of pEC_50_ values against *L. donovani* promastigotes and *T. brucei* BSF.

We further tested the seven compounds against *L. donovani* promastigotes (Figure S1), *T. brucei* bloodstream form (Figure 4) and intracellular *T. cruzi*. In the *L. donovani* promastigote growth inhibition assay, the EC_50_ values ranged from 0.009 to 7.46 μM; NVP-BGT226 was again the most potent compound (Table 1). The correlation of activity between the promastigote and amastigote assay was R^2^ = 0.64. Two structurally similar compounds, WYE-125132 and PKI-402, were 6.3- and 27.4-fold more potent against the promastigotes compared with the intracellular amastigotes (Figure 5A). In *T. brucei* assay, the compounds were generally more active and especially NVP-BGT226 had an EC_50_ value in the picomolar range (0.000054 μM). Except for sapanisertib and torkinib, all the compounds had submicromolar activities with values approximately similar to pentamidine. The extracellular forms of *L. donovani* and *T. brucei* displayed a high correlation of activity with R^2^ = 0.80 (Figure 5C), but the correlation between *T. brucei* and the amastigotes of *L. donovani* with R^2^ = 0.51 (Figure 5B). These differences may be due to genus-related differences between the parasites or to the effect of the extra host cell membrane on the penetration of compounds, or both. Furthermore, since dactolisib (NVP-BEZ235) showed varying range of in vitro activity depending on the subspecies of *T. brucei* in the previous report, the tested compounds in this study may exhibit different inhibitory activity to other subspecies of *T. brucei* or in different species of *Leishmania* [51,52]. One interesting point is the differences of antileishmanial and antitrypanosomal potency among Torin2, dactolisib and NVP-BGT225, in which the compounds share a similar structure. NVP-BGT226 and dactolisib share the same scaffold of 3-methyl-1-phenyl-8-(pyridin-3-yl)-1,3-dihydro-2*H*-imidazo[4,5-*c*]quinolin-2-one, and the corresponding structure in Torin2 is 1-phenyl-9-(pyridin-3-yl)benzo[*h*][1,6]naphthyridin-2(1*H*)-one. Since all three compounds differ by the substitutions on the phenyl and pyridine-3-yl group, the difference of potency by the sequence of NVP-BGT226 > Torin2 > dactolisib potentially have resulted from such substitutions [53,54,55,56]. The group led by Pollastri has shown that the corresponding substitutions can improve the activity of dactolisib against the in vitro growth of *T. brucei* as well as SI [37]. The same set of compounds was tested using a *T. cruzi*-infected human osteosarcoma cell line (U2OS) model. However, most of the compounds had high toxicity against the host, with a low selectivity index (data not shown). Based on the activity correlations, the compounds are expected to directly act on the parasite and share a similar mechanism of action or a target between different parasites.

### 2.4. In Vivo Efficacy of mTOR/IP3K Inhibitors in VL Mouse Model

**Figure 6:**
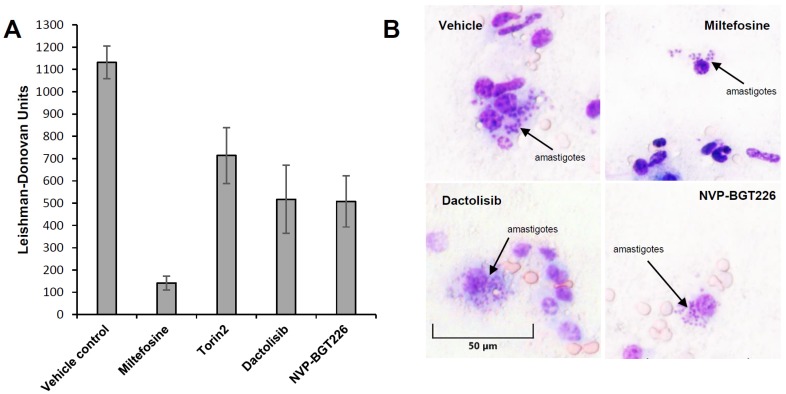
Activity of miltefosine and mTOR/PI3K compounds in *L. donovani*-infected BALB/c mice. Animals were infected and treated as described, and numbers of LDUs on slides of liver smears were counted. All treatments began 7 days after infection. The results are expressed as mean ± standard deviation values. (**A**) Groups of animals (five mice per group) were treated with single 30 mg/kg doses of miltefosine, a vehicle control with PBS, or with Torin2, dactolisib, NVP-BGT226 formulations at 15, 50, and 5 mg/kg, respectively, for 5 days via the oral route. (**B**) Postmortem Giemsa-stained liver smears were obtained from mice after no treatment or exposure to the vehicle, miltefosine, or mTOR/PI3K compounds at the doses indicated.

Commonly used animal models for evaluating compound efficacy in vivo are BALB/c (for acute) and hamster (for chronic) models [57,58,59,60,61,62]. For this proof of concept in vivo study, *L. donovani* infected BALB/c model was used to evaluate the ability of compound to reduce parasites in liver. Based on their EC_50_ and SI values from the intracellular *Leishmania* assay, we selected Torin2, dactolisib, and NVP-BGT226 (Table 1). Prior to the efficacy evaluation, the doses were selected based on the results of a literature search [45,63,64]. And in pilot toxicity study in mice all the compounds were well tolerated without displaying any overt signs of toxicity during the five consecutive days of treatment. Using the same dosing scheme, the efficacies of the compounds were evaluated in VL mouse model. Miltefosine-treated mice, showed 87 ± 2.13% inhibition of parasitemia in liver compared to the vehicle-treated control mice. In the test groups, significant decreases in parasitemia by 54 ± 3.18%, 53 ± 9.50%, and 35 ± 5.81% in the NVP-BGT226-, dactolisib-, and Torin2-treated mice, respectively (Table 2 and Figure 6), were observed and the weight of spleens were similar to that of miltefosine-treated control mice (Figure S2). In the Giemsa-stained liver smear, residual parasites not cleared by the tested compounds were observed inside host cells (Figure 6B). Even though some activity was present, the tested compounds did not show comparably improved inhibition over miltefosine at a given dose. Another point to mention is the dose used for this study. In the in vitro assay, NVP-BGT226 showed CC_50_ value of 2.66 μM, but no sign of toxicity was observed in vivo at 5 mg/kg dose. Since the dose was selected based on the previously reported literature that does not show adverse effect, the toxic effect by the compound was not observed in vivo [64]. For more thorough investigations, the maximum tolerated dose of compounds in infected animals can be determined to test efficacy of compounds with varying range of doses.

### 2.5. In Vivo Efficacy of NVP-BGT226 in T. brucei Mouse Model

**Figure 7:**
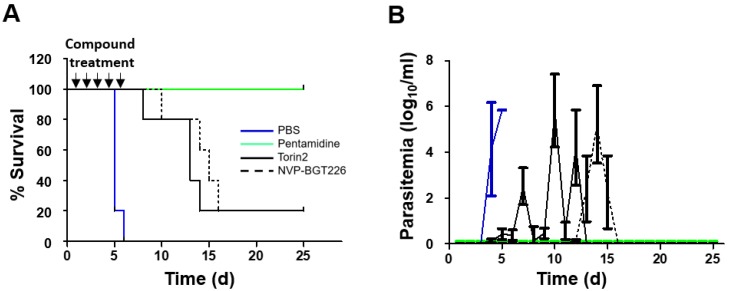
In vivo results for *T. brucei* mouse model of infection. (**A**) Mouse survival after treatment with 30 mg/kg pentamidine (solid green line), 15 mg/kg Torin2 (solid black line), 5 mg/kg NVP-BGT226 (dotted black line), or the PBS control (solid blue line) for 5 days. (**B**) Parasitemia as a function of time after treatment.

The Torin2 and NVP-BGT226 compounds had more potent activities compared with pentamidine and were thus chosen for testing in *T. brucei* acute infection model. Since the efficacy of NVP-BEZ235 was evaluated in the *T. b. rhodesiense* infected mouse model in the previous work by Pollastri et al., the compound was not selected for testing in this study [37,51,65]. *T. brucei*-infected mice with absence of any treatment all died within 6 days post-infection (Figure 7A). For the drug testing, mice were treated with pentamidine (control), Torin2, or NVP-BGT226 at 30, 15, or 5 mg/kg for 5 days via the per os route, respectively, and the survival and parasitemia results are presented in Figure 7. In the group treated with NVP-BGT226, 80% survived during 14 days post-infection followed by death afterwards, and 20% of the mice survived more than 25 days without parasitemia observed. In the Torin2-treated group, 80% of the mice survived for at least 13 days after infection, but parasitemia was observed still by day 5. NVP-BGT226 exhibited slightly improved efficacy over the Torin2-treated group with extend 1–2 days delay in survival. In addition, compared with the previous reports of NVP-BEZ235 [38], NVP-BGT226 exhibited somewhat improved efficacy in the *T. brucei* acute mouse infection model.

### 2.6. Prediction of Inhibitor Binding to Kinetoplastid TORs

**Figure 8:**
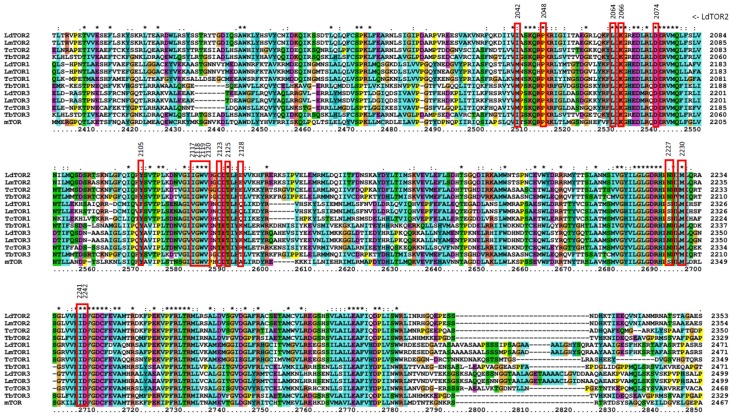
Multiple sequence alignments of the FKBP-rapamycin-binding (FRB) domains of the TOR1/2/3 kinases of *L. donovani, L. major, T. cruzi,* and *T. brucei,* and human mTOR. Seventeen amino acid residues around binding sites are indicated using red squares.

**Figure 9:**
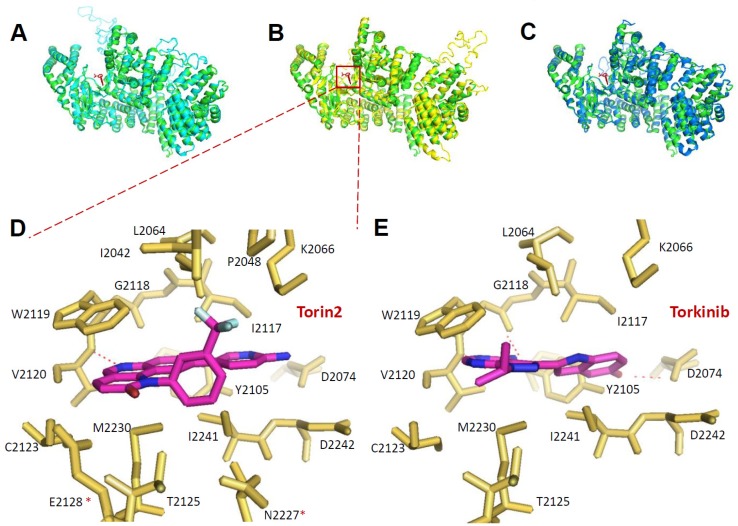
Modeling of *L. donovani* TOR domains. (**A**–**C**) Structural alignment of *Ld*TOR1/2/3 and mTORDeltaN-mLST8-Torin2 using PyMOL. The blue cartoon represents the structure of the *Ld*TOR1, the yellow cartoon represents the structure of the *Ld*TOR2, and the cyan cartoon represents the structure of the *Ld*TOR3. The green cartoon represents the structure of the mTORDeltaN-mLST8 and the red stick is the Torin2 structure. (**D**) Structures of Torin2 inhibitor bound to the *Ld*TOR2 catalytic cleft of *L. donovani*. Stick representation of Torin2 (C, red; N, blue; F, green) and of *Ld*mTOR2 residues within 4 Å (except for Asp^2074^ and Asp^2242^). Red-dotted lines indicate atoms within hydrogen-bonding distance. (**E**) Torkinib-*Ld*mTOR2 structure, represented as in (**D**), with stick representation of torkinib.

We then sought to further investigate the structural aspects of inhibitor binding. First, multiple sequence alignment analysis was performed between mTOR and kinetoplastid parasite TORs (Figure 8). In the case of *L. donovani, Ld*TOR1 showed sequence identity of 39.77% to mTOR, followed by *Ld*TOR2 at 36.77% and *Ld*TOR3 at 32.08%. *L. major*, *T. brucei*, and *T. cruzi* TOR1, 2, and 3 had similar sequence identity to mTOR within the range of 30%–40% (Table 3). Then we build homology models of kinetoplastid TORs and compared the structural similarity with human mTOR using root mean square deviation (RMSD) values. *Ld*TOR1, 2, and 3 had RMSD values of 0.374, 0.428, and 0.291, respectively, with the mTOR structure implying high degrees of structural similarity to the human mTOR structure (Table 3 and Figure 9A–C). Since *Lm*TOR3 was found not essential for the survival of the parasites in the promastigote stage, further analysis of Torin2 and torkinib binding to *Ld*TOR1 and 2 was performed by overlaying *Leishmania* TOR models onto inhibitor-mTOR structures [43]. Seventeen amino acid residues were located within 4Å of Torin2, and among them, 15 residues were found identical to that of human mTOR, except for the difference of Ala^2248^ to Glu^2128^ and Ser^2342^ to Asn^2227^ in *Ld*TOR2. In terms of molecular interaction, a hydrogen bond of N in the tricyclic benzonaphthyridinone ring of Torin2 to the O of W^2119^ backbone (2.9 Å) was identified (Figure 9D). For torkinib, the -OH and –NH_2_ moieties were predicted to interact with D^2074^ and the backbone of G^2118^, respectively (Figure 9E). These interactions were also found in the mTOR structures, which suggests a high possibility of *Ld*TOR1/2 inhibition by the inhibitors with similar binding modes.

In summary, using high-throughput screening, we identified seven mTOR/PI3K inhibitors with potent activity against the trypanosomatid parasites, *L. donovani* and *T. brucei*. Among these compounds, NVP-BGT226 was the most potent in vitro and was efficacious in VL and *T. brucei* animal models. The compound binding predictions based on the structural analysis suggests possible *Ld*TOR1/2 inhibition by mTOR/PI3K inhibitors. This group of inhibitors was extensively developed for anti-cancer treatments, but currently hurdled by toxicity in human clinical trials. Due to this reason, a direct repurposing to kinetoplastids infections may not be readily feasible, but further development of inhibitors that are more selective to the kinetoplastid TORs would be possible. Another approach is utilizing the host immune response modulating properties of this class of compounds, as demonstrated by Khadem and colleagues, and further search for mTOR/PI3K inhibitors that well balance the direct and indirect killing effects. With limited number of highly potent inhibitors against *Leishmania*, these inhibitors are excellent chemical tools to decipher TOR pathways in kinetoplastid parasites which may lead to the discovery of new drug targets.

## 3. Materials and Methods

### 3.1. Ethics Statement

All animal studies were performed in strict accordance with the guidelines and principles established by the Korean Animal Protection Law (http://animalrightskorea.org). The use of animals was approved by the Institutional Animal Care and Use Committee (IACUC) of the Institut Pasteur Korea (IACUC approval number IPK-16003–3 for VL in vivo model and IPK-19002 for acute *T. brucei* in vivo model).

### 3.2. Inhibitors

The inhibitors used in this study were purchased from MedChem Express (Monmouth Junction, NJ, USA).

### 3.3. Parasite and Cell Cultures

*L. donovani* MHOM/SD/62/1S-CL2D parasites were cultured as promastigotes at 28 °C in M199 medium (Sigma-Aldrich, St. Louis, MO, USA) with 40 mM HEPES, 0.1 mM adenine, 0.0001% biotin, and 4.62 mM NaHCO_3_ supplemented with 10% fetal bovine serum (FBS, Gibco, Carlsbad, CA, USA), 100 µ/mL penicillin (Gibco), and 100 µg/mL streptomycin (Gibco). THP-1 cells (ATCC TIB-202) were cultured in RPMI-1640 medium containing 4.5 g/L glucose, 10 mM HEPES, 1 mM sodium pyruvate, and 10% FBS. The cells were maintained in tissue culture flasks (Nunc A/S, Roskilde, Denmark) in a 5% CO_2_ incubator at 37 °C. The *T. b. brucei* Lister strain 427 (bloodstream form: BSF) was cultivated in HMI-9 medium supplemented with 10% FBS, 100 µg/mL penicillin, and 100 µg/mL streptomycin at 37 °C and a 5% CO_2_ atmosphere. The parasites were sub-cultured every 3 or 4 days and were maintained for 10 passages.

### 3.4. Screening of Bioactive Compounds Against Intracellular Leishmania

PMA-treated THP-1 human monocytic cells were seeded at 0.8 × 10^4^ cells per well in a 384-well culture plate (Greiner Bio-One, Kremsmünster, Austria) in RPMI-1640 complete medium supplemented with 10% FBS. After 48 h of incubation at 37 °C in the presence of 5% CO_2_, the promastigotes of *L. donovani* that were incubated with lectin for 30 min at 28 °C were added to the cells at a parasite to cell ratio of 20:1. Infected THP-1 cells were treated with amphotericin B (at 4 μM, positive control), miltefosine (at 10 μM, positive control), and screening compounds (at 10 μM) [66,67,68]. The negative control consisted of THP-1 infected with the parasite with only 0.5% DMSO. After 72 h, the cells that were infected and treated with the drug were washed with serum-free RPMI-1640 medium. The cells and parasites were stained using 5 μM DRAQ5 and 4% PFA. The images were acquired based on reading using an Operetta^®^ automated microscope (PerkinElmer, Inc., Waltham, MA 02451 USA). They were further analyzed using Columbus^TM^ (PerkinElmer, Inc. Waltham, MA, USA) software to quantify parasite numbers, host cell numbers, and infection ratios. In brief, large-sized nucleus of host cells was first detected using Draq-5 (Thermo Fisher, Rockford, IL, USA) signal and the host cell boundary masking was performed using the low-intensity signals from cytosols (additional feature of Draq-5). Then the small-sized nucleus signal by Draq-5 was used to identify parasites within the area of masked host cell. Infection ratio (IR) was determined with the value of the number of infected cells divided by total number of cells and the average number of parasites per macrophage (P/φ) was defined by the value of the number of parasites divided by the number of infected cells in the acquired image. The average IR value of the negative control wells was calculated as 0.53. Compounds selected based on the screening results were further assessed in a dose-dilution manner (two-fold serial dilution for 20 points starting from 100 μM) using the same method.

### 3.5. Parasite Growth Inhibition

*L. donovani* promastigote and *T. b. brucei* Lister 427 BSF growth inhibition were assayed by measuring the conversion of resazurin to resorufin. The assays were performed in 384-well plates that were seeded with *L. donovani* promastigotes or *T. brucei* 427 BSF (5 × 10^4^ cells per well). After seeding, the parasites were exposed to the compounds for 3 days. Resazurin sodium salt (200 μM; R7017; Sigma-Aldrich, St. Louis, MO, USA) was then added, and the samples were incubated for 5 h. After incubation, the parasites were fixed using 4% paraformaldehyde, and the plates were analyzed using a Victor3^TM^ plate reader (PerkinElmer, Inc., Waltham, MA, USA) at 590 nm (emission) and 530 nm (excitation) [69]. Amphotericin B and miltefosine were used as the reference drugs for the *L. donovani* promastigote growth inhibition [67]. Pentamidine was used for the *T. b. brucei* Lister 427 BSF growth inhibition assay [68].

### 3.6. In Vivo Experiments

#### 3.6.1. VL Mouse Model

Groups of five-week-old female BALB/c mice (five per group) were injected with 2 × 10^7^ hamster spleen-derived *L. donovani* amastigotes via the retro-orbital venous sinus route. From day 7 post-infection, groups of mice were treated using the drug vehicle only, miltefosine (30 mg/kg), Torin2 (15 mg/kg), dactolisib (50 mg/kg), or NVP-BGT226 (5 mg/kg). The highest dose for each compound without showing adverse effects was selected based on literature search [44,45,49]. The vehicle for the compounds was 5% *N*-methylpyrrolidone, 15% polyvinylpyrrolidone, and 80% deionized water. Drug dosing solutions were freshly prepared each day. All drugs were administered once daily for 5 days via the per os route. On day 16 post-infection, all animals were humanely euthanized and assessed microscopically using Giemsa-stained liver imprints. Parasite burdens were measured by counting (blinded to treatment) the number of amastigotes per 1000 cell nuclei and multiplying this number by the liver weight (mg) (Leishman-Donovan Unit: LDU) [70]. The LDU values for the drug-treated samples were compared to those of the untreated samples, and the percent inhibition values were calculated.

#### 3.6.2. HAT Mouse Model

BALB/c mice were infected with *T. b. brucei* Lister 427 (4 × 10^4^ cells) by intraperitoneal (*i.p.*) injection. The mice were divided into groups (*n* = 5), and drug treatment was performed for five consecutive days by starting from day 1 post-infection administering 30 mg/kg, 15 mg/kg, and 5 mg/kg of pentamidine, Torin2, and NVP-BGT226, respectively. Parasitemia was evaluated daily for 2 weeks by blood collection from the mouse tail vein, and survival was monitored for 1 month. Mice showing impaired health status and/or with a parasite load > 10^8^ cells per mL of blood were euthanized.

### 3.7. Prediction of Compound Binding Modes

The protein sequence for *L. donovani* TOR1 (*Ld*BPK_366580.1), *Ld*TOR2 (*Ld*BPK_344160.1), *Ld*TOR3 (*Ld*BPK_343750.1), *L. major* TOR1 (*Lmj*F.36.6320), *Lm*TOR2 (*Lmj*F.34.4530), *Lm*TOR3 (*Lmj*F34.3940), *T. brucei* TOR1 (*Tb*427.04.800), *Tb*TOR2 (*Tb*427.04.420), *Tb*TOR3 (Tb927.4.800), *T. cruzi* TOR1 (BCY_11356), *Tc*TOR2 (BCY_20486), *Tc*TOR3 (BCY_20528), and mTOR (NP_004949) were downloaded from the Kinetoplastid Genomic Resource database (https://tritrypdb.org/tritrypdb/) and the Proteins database (https://www.ncbi.nlm.nih.gov/). The sequence alignment was performed using ClustalX2.1 (Cambridgeshire, UK). The homology models of LdTOR1/2/3 were obtained using SWISS_MODEL (http://swissmodel.expasy.org) with the Torin2 bound mTOR structures (PDB ID: 4JSX, and 4JT5, http://www.rcsb.org) as templates [71,72]. The PyMOL 1.3 program (Palo Alto, CA, USA) was used to obtain the structural alignment results.

### 3.8. Statistical Analyses

All of the half maximal effective concentrations (EC_50_) and the half maximal cytotoxic concentrations (CC_50_) values were calculated using two independent experiments. The dose-response curves were fitted using GraphPad Prism 6 software (GraphPad Software, San Diego, CA, USA) by using a sigmoidal dose-response equation with a variable hill slope option.

## Figures and Tables

**Table 1:** Efficacy of tested compounds in THP-1 cells infected with intracellular *L. donovani, L. donovani* promastigote growth; and *T. brucei* bloodstream form.

Compounds	Intracellular *L. donovani* EC_50_ ± SD ^a^ (μM)	THP-1 Cell CC_50_ ± SD ^a^ (μM)	Selectivity Index (SI)	*L. donovani* Promastigote EC_50_ ± SD ^a^ (μM)	*T. brucei* Lister 427 BSF EC_50_ ± SD ^a^ (μM)
Miltefosine	4.81 ± 0.81	>40	>8.3	1.03 ± 0.63	NA
Amphotericin B	0.34 ± 0.04	>4	>11.8	0.015 ± 0.003	NA
Pentamidine	NA	NA	NA	NA	0.15 ± 0.01
Torin2	0.25 ± 0.03	30.50 ± 1.3	122	0.03 ± 0.004	0.11 ± 0.01
Dactolisib (NVP-BEZ235)	0.36 ± 0.07	>100	≥277.8	0.38 ± 0.06	0.12 ± 0.01
NVP-BGT226	0.14 ± 0.02	2.66 ± 0.46	19	0.009 ± 0.001	0.000054 ± 0.000003
Sapanisertib	6.39 ± 1.12	>100	≥39	7.46 ± 1.04	5.34 ± 0.98
Torkinib	3.83 ± 0.98	33.43 ± 1.4	8.7	3.86 ± 1.11	8.16 ± 1.52
WYE-125132	2.26 ± 0.71	43.27 ± 2.7	19	0.36 ± 0.04	0.12 ± 0.01
PKI-402	13.44 ± 2.35	28.48 ± 1.2	2.1	0.49 ± 0.22	0.26 ± 0.02

^a^ Shown are mean half maximal effective concentrations (EC_50_) ± SD (standard deviations) of data from duplicate measurements; NA, not applicable; Selectivity index (SI = THP-1 CC_50_/Intracellular *L. donovani* EC_50_).

**Table 2:** Efficacy of tested compounds in *L. donovani*—infected BALB/c mice.

Compounds	Dose (mg/kg)	LDU in the Liver (Mean ± SD)	Inhibition of *L. donovani* In Vivo (%) (Mean ± SD)
Vehicle	−	1132 ± 73	0
Miltefosine	30	142 ± 24	87 ± 2.13
Torin2	15	735 ± 66	35 ± 5.81
Dactolisib (NVP-BEZ235)	50	527 ± 108	53 ± 9.50
NVP-BGT226	5	516 ± 36	54 ± 3.18

Parasite burden (LDU, Leishman-Donovan Unit) was evaluated 7 days after compound administration. Data are presented as mean ± SD; NA, not applicable.

**Table 3:** The multiple sequence alignment between mTOR and kinetoplastid parasite TORs.

	mTOR	*L. donovani*			*L. major*			*T. cruzi*			*T. brucei*		
		1	2	3	1	2	3	1	2	3	1	2	3
Sequence identity (%)	100	39.77	36.77	32.08	39.83	36.96	32.18	38.19	36.23	33.63	33.47	35.15	34.78
RMSD	0	0.374	0.428	0.291	0.157	0.296	0.280	0.360	0.393	0.392	0.284	0.173	0.422

Sequence identity (%) and root mean square deviation (RMSD) were calculated by using SWISS MODEL and PyMOL, respectively.

## Supplementary Materials

The following are available online, Figure S1: Dose-response curves of miltefosine, amphotericin B, and mTOR/PI3K compounds against *L. donovani* promastigote growth, Figure S2: Changes in spleen weight in *L. donovani*-infected BALB/c mice and effects of treatment with compound, Table S1: The list of 1742 compounds, Table S2: The relationships between mTOR/PI3K inhibition activity and intracellular *L. donovani* inhibition activity.
